# Participatory research approaches in long-term care facilities for older adults: a meta-ethnography

**DOI:** 10.1080/17482631.2024.2431449

**Published:** 2024-11-24

**Authors:** Sophie Nadia Gaber, Manuel Guerrero, Lena Rosenberg

**Affiliations:** a Department of Neurobiology, Care Sciences and Society, Division of Occupational Therapy, Karolinska Institutet, Stockholm, Sweden; b Department of Rehabilitation, School of Health and Welfare, Jönköping University, Jönköping, Sweden; c Department of Women’s and Children’s Health, Healthcare Sciences and e-Health, Uppsala University, Uppsala, Sweden; d Centre for Research Ethics and Bioethics, Uppsala University, Uppsala, Sweden; e Department of Bioethics and Medical Humanities, Faculty of Medicine, University of Chile, Santiago, Chile

**Keywords:** Aged, co-creation, co-production, collaboration, community-based participatory research, dementia, meta-ethnography, qualitative synthesis, nursing homes, long-term care facilities

## Abstract

**Purpose:**

There is growing interest in the potential of participatory research approaches to democratize research, empower participants and contribute to targeted health and social care. Participatory research approaches are emphasized in ethical and funding applications regarding patient and public involvement; however, less is known about their use in long-term care facilities for older adults. This meta-ethnography seeks to provide an increased understanding and novel conceptualization of participatory research approaches in long-term care facilities for older adults.

**Methods:**

A meta-ethnography was used to synthesize qualitative literature on participatory research approaches in long-term care facilities for older adults. In total, 1,736 articles were screened at title and abstract level, 35 studies were eligible for full-text review and 10 articles were included.

**Results:**

The following third-order constructs were identified as salient to the conceptualization and use of participatory research approaches in long-term care facilities for older adults: 1) participatory backdrops, 2) collaborative places, 3) seeking common ground and solidarity, 4) temporal considerations, and 5) empowerment, growth, and cultural and social change.

**Conclusion:**

This meta-ethnography contributes a novel conceptualization and six recommendations to enhance the design and implementation of participatory research approaches as democratic spaces of exchange and collaboration for older adults.

## Introduction

In recent times, concerns have been raised about the quality of life and care among older adults living in long-term care facilities (LTCFs) (Mondaca et al., 2018; Shura et al., 2011). Concerns relate to systematic institutionalization and medicalization of older adults (Corazzini et al., 2016; de Medeiros et al., 2020; Mondaca et al., 2018) with less attention given to the potentiality of everyday life to provide opportunities for lifelong learning (de Medeiros et al., 2020; Kydd & Fulford, 2020), participation in activities (Fallahpour et al., 2022; Gustavsson et al., 2015), and social engagement (Mondaca et al., 2018; Shura et al., 2011). Earlier research highlights the importance of communication and collaboration between different social and organizational actors for facilitating engagement in everyday activities among older adults in LTCFs (Fallahpour et al., 2022; Gustavsson et al., 2015; Wood et al., 2009), but strategies for how to do this remain unclear. Potential strategies include participatory research approaches which provide opportunities to help inform and develop health and social care (Cargo & Mercer, 2008; Vaughn & Jacquez, 2020), including LTCFs which are targeted to the care needs of older adults as well as persons working and interacting with the wider LTCF community. LTCF is used as an umbrella term for different types of accommodation where older adults live (e.g, nursing homes, assisted living facilities, residential facilities, and care homes), that can provide services (i.e., medical and assistive care) to those older adults living in the LTCFs (World Health Organization, 2022). LTCFs vary between and within countries, however, they do not include home-based long-term care, or community-based services (e.g., community centers, respite care, or adult day care) (World Health Organization, 2022). The rationale for focusing on LTCFs was to explore participatory research approaches among individuals and communities of older adults who live on-site and their interactions and opportunities for collaboration with staff and other actors.

Participatory research approaches are defined as research designs, methods, and conceptual frameworks that involve systematic inquiry in collaboration with persons who are directly involved and invested in the research and research outcomes, and which emphasize social action and change (Cargo & Mercer, 2008; Levac et al., 2019; Vaughn & Jacquez, 2020). This working definition served as a departure point to explore participatory research approaches which have been scarcely researched in the context of LTCFs. The term “approaches” rather than “methods” was chosen to acknowledge the ambiguity and breadth of participatory research, for example co-creation and co-design (Leavy, 2017) can be used to address design problems, whereas advocacy and community-orientated approaches such as community-based participatory research (CBPR) (Lake & Wendland, 2018; Leavy, 2017) and participatory action research (PAR) (Benjamin-Thomas et al., 2018; Hand et al., 2019) may emphasize democratization and empowerment of marginalized individuals and communities (Lake & Wendland, 2018; Leavy, 2017). Participatory research approaches encompass diverse theoretical and philosophical influences that are harnessed through the person’s lived experiences (Benjamin-Thomas et al., 2018; Reason & Bradbury, 2008), and a shared philosophy of doing research “with” rather than “on” persons (Cargo & Mercer, 2008; Vaughn & Jacquez, 2020). Inspired by scholars of the Global South, such as Freire (Freire, 1993) and Fals Borda (Reason & Bradbury, 2001), the underlying ontological view is that persons have the potential to make changes in their own lives through reflection, creativity, and praxis (Kindon et al., 2007; Lake & Wendland, 2018; Reason & Bradbury, 2001). This may be achieved through the opening up of communicative spaces, both physical and social, for the exchange of knowledge and dialogues (Benjamin-Thomas et al., 2018; Habermas, 1984).

Among the few studies in LTCFs that are described as participatory research, older adults have tended to be involved only as “subjects” or in a more distanced advisory role (Blair & Minkler, 2009; Smith et al., 2017). Despite good intentions, participants may experience “epistemic injustice” – a concept that the philosopher and feminist Miranda Fricker (Fricker, 2007, 2013) describes as occurring when personal narratives are perceived as not worth being listened to and a person feels his/her knowledge is undervalued (Groot et al., 2022). “Epistemic injustice” is exacerbated by expectations that participatory research will involve increased opportunities for a democratic sharing of knowledge and power compared with conventional research (Blair & Minkler, 2009; Smith et al., 2017). Participatory research approaches are especially relevant for marginalized communities (Fals Borda, 2001; Levac et al., 2019) and have the potential to address ageist and stigmatizing assumptions about older adults’ interests and capabilities by providing opportunities for them to express their interests, preferences, and needs (Smith et al., 2017). Thus, there is a need to explore and problematize what it means to involve actors (i.e., older adults, staff, management, volunteers) in LTCFs who have little to no prior research experience and training (Lake & Wendland, 2018), how sustainable these participatory research approaches are (Benjamin-Thomas et al., 2018), and how researchers (sometimes referred to as practitioners) can prevent their research from becoming a tokenistic or “tick-box” exercise to acquiesce with grant funders’ or ethical boards’ calls for greater patient and public involvement (Benjamin-Thomas et al., 2018; Hand et al., 2019).

In summary, there are earlier systematic reviews that have contributed insights regarding uses of participatory research approaches with older adults, for example, general impacts of older adults’ patient and public involvement in health and social care (Baldwin et al., 2018), or good practices, facilitators, and barriers to active involvement of people living with dementia and long-term care users (Groothuijse et al., 2024), and the specific role of older care home residents as collaborators or advisors in research (Backhouse et al., 2016). However, there has been limited conceptualization of participatory research approaches in LTCFs for older adults which is needed to better understand these approaches, and how they can enhance the health and well-being of older adults in LTCFs, including potential opportunities to address epistemic injustices. This motivates the need to conduct a scholarly inquiry and contextualization of the topic, by synthesizing both conceptualizations and uses of participatory research approaches across different studies, or regarding specific contexts and target populations (Benjamin-Thomas et al., 2018) using a meta-ethnography. Increased understanding of the enabling characteristics associated with specific contexts, namely LTCFs, is needed to better support the design and implementation of participatory research approaches in LTCFs for older adults.

## Aim and research questions

The aim of this study was to provide an increased understanding and novel conceptualization of participatory research approaches in LTCFs for older adults. The research questions were as follows:

1. What are the characteristics of participatory research approaches that enable participation of older adults, staff, and other actors in LTCFs for older adults?
2. How are participatory research approaches addressed by researchers, older adults, staff, and other actors in LTCFs for older adults?

## Methods

### Design

This meta-ethnography builds on Noblit and Hare’s (Noblit & Hare, 1988) framework for qualitative synthesis, as well as more recent refinements for meta-ethnography proposed by Cahill et al. (2018), France et al. (2019), and Sattar et al. (2021). This meta-ethnography followed the seven phases proposed by France et al.'s (2019) eMERGe guidelines: 1) selecting the meta-ethnography and getting started (i.e., describing the background rationale and context for the meta-ethnography), 2) identification of relevant studies, 3) reading and data extraction, 4) determining how the studies are related, 5) translating the studies into one another, 6) synthesizing translations, and 7) expressing the synthesis.

#### Phase 1—selecting the meta-ethnography and getting started

We chose to perform a meta-ethnography based on its defining features as an interpretative and inductive approach to synthesize qualitative primary research studies (Brookfield et al., 2019; France et al., 2019; Sattar et al., 2021), and its potential to broaden our findings because according to Noblit and Hare (1988, p. 13) it “seeks to go beyond single accounts to reveal the analogies between the accounts”. Thus, we sought to construct a novel interpretation based on a diverse selection of qualitative studies. This differs from aggregative synthesis methodologies (e.g., thematic analysis), or those that combine qualitative and quantitative data (e.g., critical interpretive synthesis, realist synthesis) (Brookfield et al., 2019; France et al., 2019). A defining feature of meta-ethnography is that researchers seek to understand and re-interpret conceptual data (i.e., meaning, concepts, and contexts) of the studies that they are synthesizing through the dual processes of translation and synthesis (Sattar et al., 2021), as described in the subsequent Phases 5, 6, and 7.

#### Phase 2—search strategy and identification of relevant studies

To promote transparency and avoid duplication, a protocol describing the search strategy was registered in the International Prospective Register of Systematic Reviews (PROSPERO) (registration number: CRD42021275187). Table 1 outlines how the literature search was conducted according to the STARLITE guidelines (Booth, 2006).

**Table 1.** Literature search strategy.

S: Sampling strategy	With support from scientific librarians, we (SNG, MG, LR) conducted a *comprehensive* search of five databases relevant to the field of interest (i.e., from medicine and health care, education, sociology, and social science fields). Next, we refined our search strategy *purposively* through our inclusion and exclusion criteria as well as sampling decisions e.g., to include studies that specifically related to participatory research approaches in LTCFs, and to exclude studies with concepts unrelated to participatory research approaches, and settings or populations that are unrelated to LTCFs. We also screened studies for conceptual richness of data.	
T: Type of study	Any type of qualitative study (e.g., interviews, focus groups, observational).	
A: Approaches	With support from scientific librarians, we performed a comprehensive, literature search in July 2021. The search was updated in June 2022 and November 2023 based on the method described by Bramer and Bain (2017) repeating the searches and deduplicating against the previous search (Manchha et al., 2023). The reference lists of the studies selected for full-text review were also hand-searched for additional relevant publications.	
R: Range of years (start date: end date)	There have been considerable advances in participatory research approaches within care and research of older adults in the last two decades. This has partly been motivated by the increased interest and formalization of patient and public involvement in research and funding applications (Snoeren et al., 2016; Woelders & Abma, 2019). Thus, to ensure that the included studies are relevant to shifting research and policies concerning older adults, only studies published between 2001 and 2023 were included.	
L: Limits	No geographic limiters were used as we were interested in examples of participatory research approaches with older adults living in LTCFs and/or staff working in LTCFs from different countries, cultures, and contexts.	
I: Inclusion and exclusions	*The inclusion criteria were*: publication in English; peer-reviewed publications; qualitative design; specifically related to participatory research approaches with older adults living in LTCFs and/or staff (but not students) working in LTCFs. We referred to the working definition of participatory research approaches described in the introduction. *The exclusion criteria were*: quantitative or mixed methods designs; book chapters, theses, commentaries, discussion papers, or review articles; concepts unrelated to participatory research approaches (based on the aforementioned working definition), and settings or populations that are unrelated to LTCFs for older adults.	
T: Terms used	A block search strategy was used based on the following PICo (population, phenomena of interest, and context) framework and keywords:	
	Population	Dementia; Alzheimer*; Alzheimer disease; cognitive impairment; older adult*; older person*; older people; aged, 80 and over; elder*; aged; older men; older man; older women; older woman; older female*; older resident*; elderly resident* Staff; care providers; nurses; nursing assistants; nurse assistants
	Phenomena of interest	Collaborat*; collaborat* research; collaborat* methods; collabor* approach*; co-creat*; cocreat*; co-design; codesign*; co-produc* coproduc*; co-research*; coresearch*; participat*; participatory method*; participatory research; participatory action research; action research; visual method*
	Context	Nursing home; care home*; long term care; residential care facilit*; aged care facilit*; residential home; old people* home; elder care; assisted living facilities; residential facilities; homes for the aged
E: Electronic sources	MEDLINE (Ovid); the Cumulative Index of Nursing and Allied Health Literature [CINAHL] (Ovid); the Education Resources Information Center [ERIC]; Sociological Abstracts; and Web of science.	

Two of the authors (SNG, LR) independently screened the database search results at the title and abstract level for relevance to the aim, research questions as well as the inclusion and exclusion criteria, using the Rayyan.ai bibliometric and review software (Ouzzani et al., 2016). Any disagreements were discussed with the third author (MG) to reach consensus. The reference lists of the studies selected for full-text review were also hand searched for additional relevant publications. Ten publications were considered appropriate for further review and the subsequent full-text readings focused on elucidating concepts.

### Critical appraisal

We independently appraised the full texts using an adapted version of the Critical Appraisal Skills Programme (CASP) checklist (Table 2) (Critical Appraisal Skills Programme, 2018). Following the precedent of earlier research, the CASP checklist was adapted for the purposes of our study through the addition of one question about whether concepts and/or the conceptual framework were adequately described (Malpass et al., 2009). Thus, the CASP checklist was used to appraise the value of studies for informing the qualitative synthesis and was not a determinant of inclusion or exclusion in the present meta-ethnography (i.e., none of the 10 articles were excluded based on the CASP scores). The CASP checklist enabled the reviewers to identify key features and characteristics of the 10 articles which were taken into consideration in the subsequent phases of reading and data extraction.

**Table 2.** Critical appraisal skills programme (CASP) checklist.

Literature source	Clear aims	Appropriate methodology	Appropriate design	Appropriate recruitment strategy	Appropriate data collection	Appropriate consideration to research—participant relationship	Ethical considerations	Rigorous data analysis	Clear statement of findings	Valuable research	Concepts/conceptual framework adequately described	Score
Baur & Abma, (2012)	Y	Y	Y	Y	Y	Y	Y	Y	Y	Y	Y	22/22
Buckley et al., (2018)	Y	Y	Y	Y	Y	Y	N	Y	Y	Y	Y	20/22
Hewitt, Draper & Ismail, (2013)	Y	Y	Y	Y	Y	Y	Y	Y	Y	Y	Y	22/22
Mondaca et al., (2019)	Y	Y	Y	Y	Y	Y	Y	Y	Y	Y	Y	22/22
Mondaca et al., (2020)	Y	Y	Y	Y	Y	Y	Y	Y	Y	Y	Y	22/22
Pappne Demecs & Miller, (2019)	Y	Y	Y	?	Y	Y	Y	Y	Y	Y	Y	21/22
Snoeren et al., (2016)	Y	Y	Y	Y	Y	Y	Y	Y	Y	Y	Y	22/22
Van Malderen et al., (2017)	Y	Y	Y	Y	Y	?	Y	Y	Y	Y	Y	21/22
Willis et al., (2018)	Y	Y	Y	Y	Y	Y	Y	Y	Y	Y	Y	22/22
Woelders & Abma, (2019)	Y	Y	Y	Y	Y	Y	?	Y	Y	Y	Y	21/22

Scoring: Y (yes) = 2;? (can’t tell) = 1; N (no) = 0.

#### Phase 3—reading and data extraction

We read the 10 studies several times in full to familiarize ourselves with each study and to extract data from each of the included studies. To ensure consistency between authors, a data extraction form based on Sattar et al. (2021) was developed, piloted, and the final version included: categories (primary authors’ categorization of their data), first-order constructs (participants’ quotes), second-order constructs (primary authors’ interpretations from the primary study), and our reflections as reviewers which formed the basis of our third-order constructs (reviewers’ interpretations) in the subsequent phases. To contextualize the data extraction, we referred to the study characteristics (Supplementary Table S1) and consulted the original studies to check the data for accuracy. Data were extracted from across each full primary study but with a particular focus on the findings. We extracted the data verbatim to preserve the original wording and to differentiate it from our new interpretations in subsequent stages of the meta-ethnography. Table 3 presents an extract of the form.

**Table 3.** Extract of the data extraction form.

Article	Categories	First-order constructs (participant quotes)	Second-order constructs (Primary author interpretations)	Reviewers’ reflections
Baur & Abma (2012)	Ownership and responsibility (p.1070)	“If the other residents say that the meals have improved a bit, I think: ha, that’s what we achieved! We can pat ourselves on the back. Look what we’ve managed to get done.” (p. 1070)	They were proud of themselves. This was expressed by some of these residents. They even thought up a name for themselves to emphasize their group identity: The Taste Buddies. They felt that they owned the practice improvements (p. 1070)	Pride and growing empowerment: growing empowerment and trust led to a feeling of ownership and real influence about what they had achieved. This growing pride and empowerment was reinforced by their group identity as The Taste Buddies.

#### Phase 4—determining how the studies are related

To determine how the studies were related, we independently created a list of emerging concepts, juxtaposing and comparing them across the different studies. Through ongoing discussions, we reflected upon and refined our interpretations to reach a consensus about how the studies are related. The studies were compared based on their findings (explanation of a phenomenon from the primary study accounts), in addition to their research design, conceptual and ethical approaches, and any relevant contextual factors. Disagreements were resolved through discussions with our respective research networks.

#### Phase 5—translating the studies into one another

Following guidance from Sattar et al. (2021), we met to discuss and compare concepts from each study in relation to the other studies. We created tables to identify similarities and differences between the concepts to help preserve the relationships within and across the studies. Table 4 presents the outcome of this translation process including our third-order constructs which are discussed in Phase 6.

**Table 4.** Presentation of summary definitions, second- and third-order constructs.

Summary definition (translation) of second-order constructs	Second-order constructs	Third-order constructs
Researchers observed institutionalized behaviors and a professionalization of caring relationships in LTCFs which negatively influenced older adults’ and staff members’ willingness to engage in participatory research approaches, to take risks, or to challenge themselves (Baur & Abma, 2012; Buckley et al., 2018; Hewitt et al., 2013; Mondaca et al., 2020; Mondaca et al., 2019; Snoeren et al., 2016; Woelders & Abma, 2019).	Institutional hinders	Participatory backdrops
Despite face-to-face civility, the participatory backdrops and overall atmosphere of the LTCFs were shaped by specific hierarchies, relationships, and power dynamics which may be more or less conducive to the empowerment rationale of participatory research approaches (Baur & Abma, 2012; Buckley et al., 2018; Hewitt et al., 2013; Mondaca et al., 2020; Mondaca et al., 2019; Willis et al., 2018; Woelders & Abma, 2019). However, there was a disconfirming case of positive change and empowerment of older adults being achieved when it was possible to harness the hierarchical power of the management (Woelders & Abma, 2019).	Organizational and power dynamics	
Engagement and influence were conceptualized as fragile and contingent on the conditions of the very moment and on other people to be realized (Baur & Abma, 2012; Hewitt et al., 2013; Mondaca et al., 2019; Snoeren et al., 2016; Willis et al., 2018). Assumptions, preconceptions, stigma, and stereotypes among older adults, staff, and management served as a potential resisting force for change (Buckley et al., 2018; Mondaca et al., 2020; Van Malderen et al., 2017; Willis et al., 2018).	Fragility, rigidity, and fluidity of participatory research approaches	
Participants emphasized the importance of creating a good atmosphere to enable participatory research approaches and this involved a meeting place, common area, or a “third space” (Mondaca et al., 2019; Pappne Demecs & Miller, 2019; Snoeren et al., 2016; Van Malderen et al., 2017).	Collaborative, inclusive, and safe places	Collaborative places
Spaces, persons, and objects could be a focus of participation stimulating sensory and social responses, and simultaneously the materiality of creative projects contributed new qualities to the LTCF environments (Baur & Abma, 2012; Buckley et al., 2018; Pappne Demecs & Miller, 2019). However, there was a disconfirming case of spaces, persons, and objects also as a focus of disruption (Pappne Demecs & Miller, 2019).	Materiality of creative expressions	
Participants identified topics that they perceived as important and meaningful, for participating older adults this was related to the desire to be “seen” as human beings and for staff it reflected a desire to be able to provide what they perceived as good care (Baur & Abma, 2012; Buckley et al., 2018; Hewitt et al., 2013; Mondaca et al., 2020; Mondaca et al., 2019; Snoeren et al., 2016; Willis et al., 2018; Woelders & Abma, 2019). Participatory research approaches contributed to the development of a broader conception of activities in the LTCFs (Baur & Abma, 2012; Buckley et al., 2018; Mondaca et al., 2020; Mondaca et al., 2019; Pappne Demecs & Miller, 2019; Snoeren et al., 2016; Willis et al., 2018; Woelders & Abma, 2019).	Dignifying and meaningful topics and activities	Common ground and solidarity
Older adults desired open communication and to feel informed about news and activities relevant to the LTCF community (Baur & Abma, 2012; Buckley et al., 2018; Hewitt et al., 2013; Mondaca et al., 2020; Mondaca et al., 2019; Pappne Demecs & Miller, 2019; Snoeren et al., 2016; Van Malderen et al., 2017; Willis et al., 2018; Woelders & Abma, 2019). However, there were disconfirming cases of communication being constrained based on perceived privacy and ethical concerns (Hewitt et al., 2013; Woelders & Abma, 2019), or the issue of sharing personal experiences which may also involve reliving narratives of exclusion and traumatic memories which required appropriate support (Willis et al., 2018).	Open communication and feeling informed	
The participatory research projects were characterized by their slow-paced progression, initial hesitancy, turning points, stagnation and/or change. There were various reasons for initial hesitancy including, curiosity, creative anticipation, and the perceived need to wait for the “right time” to set them in motion (Mondaca et al., 2020; Pappne Demecs & Miller, 2019; Snoeren et al., 2016). However, there was a disconfirming case that prevented initial participation based on the perceived cultural norm that it was “not chic” to complain and that it was important to be grateful (Baur & Abma, 2012).	Initial hesitancy, turning points, stagnation and/or change	Temporal considerations
Participants expressed and enacted their agency by choosing when and how they wanted to participate in the participatory research project, expressing a sense of loss at the end of the project, but it was unclear what happened after the participatory research project in terms of future plans and sustainability of changes (Baur & Abma, 2012; Buckley et al., 2018; Hewitt et al., 2013; Mondaca et al., 2020; Mondaca et al., 2019; Pappne Demecs & Miller, 2019; Snoeren et al., 2016; Van Malderen et al., 2017; Willis et al., 2018; Woelders & Abma, 2019). However, there was a disconfirming case involving only one study which explicitly discussed future plans and sustainability of the participatory research approaches at the end of the project (Snoeren et al., 2016).	Future plans and sustainability	
Older adults were initially cautious about expressing personal opinions, but they gradually became energized and compelled by their responsibility to stand up for other older adults and a sense of relational empowerment that they were doing it for the greater good of the LTCF community (Baur & Abma, 2012; Mondaca et al., 2020; Mondaca et al., 2019; Pappne Demecs & Miller, 2019; Snoeren et al., 2016; Willis et al., 2018; Woelders & Abma, 2019). Participatory research approaches provided the circumstances for the germ of individual empowerment and personal growth to flourish as participants in gained self-confidence and were better able to express themselves (Hewitt et al., 2013; Pappne Demecs & Miller, 2019; Snoeren et al., 2016; Willis et al., 2018).	Relational and individual empowerment and growth	Empowerment, growth, and cultural and social change
Changes in relationships occurred as when the perceptions and motivations for participatory research shifted from an instrumental focus towards an increased value of dialogue, seeking a common ground, and a “we” feeling among the LTCF community (Baur & Abma, 2012; Buckley et al., 2018; Mondaca et al., 2020; Mondaca et al., 2019; Pappne Demecs & Miller, 2019; Snoeren et al., 2016; Van Malderen et al., 2017; Willis et al., 2018; Woelders & Abma, 2019).Shifts in attitudes, values, and culture were attributed to collective actions within the LTCF that would not have previously occurred (before the project) and this required time to change (Baur & Abma, 2012; Mondaca et al., 2020; Mondaca et al., 2019; Snoeren et al., 2016; Willis et al., 2018; Woelders & Abma, 2019).	Changes in roles, relationships, and culture	

#### Phase 6—synthesizing translations

Phase 6 involved synthesizing translations as well as interpreting and constructing our novel conceptualization (line-of-argument) (France et al., 2019). To synthesize translations and develop the third-order constructs (Table 4), our concepts and quotes from the studies were analyzed and interpreted together, and we reflected upon alternative interpretations before reaching a consensus. During this phase, the individual studies were viewed as a whole (Sattar et al., 2021). Utilizing an inductive approach, we independently synthesized the data from the studies by integrating the reciprocal and refutational translations and merging the findings to create an overarching conceptualization (line-of-argument) which was expressed narratively and as a conceptual model (Figure 2) (France et al., 2019; Noblit & Hare, 1988).

#### Phase 7—expressing the synthesis

**Figure 1.**
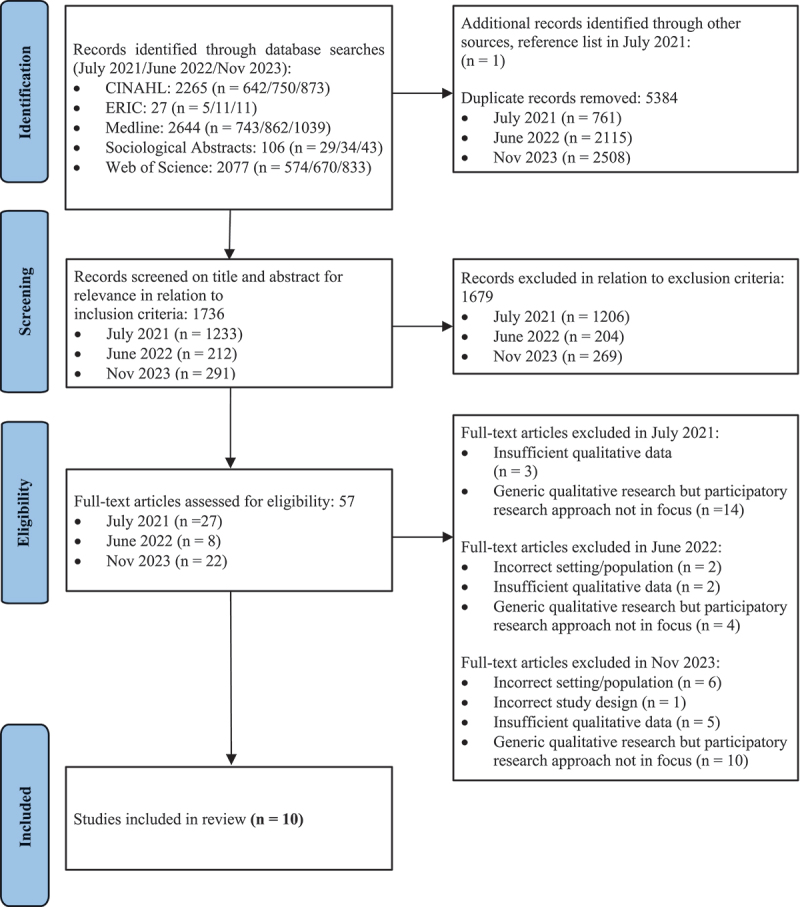
PRISMA flow diagram.

In addition to the eMERGe reporting guidelines (France et al., 2019), we followed the Enhancing Transparency in Reporting the Synthesis of Qualitative Research (ENTREQ) statement (Tong et al., 2012) and presented the search outcome in a flow diagram based on the PRISMA-updated guidelines for reporting systematic reviews (Figure 1) (Page et al., 2021). In Phase 7, we discussed the main findings and reflected upon strengths, limitations, and aspects of reflexivity in this study. Through this process, we developed recommendations to support our novel conceptualization of participatory research approaches in LTCFs. The proposed meta-ethnography plan and initial findings were presented orally at a public health scientific meeting in November 2023 (prior to the third updated literature search) (Gaber et al., 2023). Following the scientific meeting, we performed an updated literature search and feedback from the presentation was used to refine and develop the present study.

#### Reflexivity

The interdisciplinary review team are from three countries (England, Chile, and Sweden) and collectively we brought a diversity of perspectives, expertise, and experiences from occupational therapy, ethics, sociology, and architecture. While the review team members are not older adults themselves, they have extensive experience working with older adults using participatory research approaches across different contexts, including LTCFs. This prior experience motivated the decision to use a meta-ethnography to synthesize qualitative studies regarding conceptualizations and uses of participatory research approaches with a view to enhancing the design and implementation of participatory research approaches in LTCFs for older adults. We acknowledge that it may have been beneficial to involve older adults in the review process (i.e., to discuss and reflect on different interpretations); however, it was not feasible for the current meta-ethnography. It may be possible to involve older adults in future research and practice, by collaborating with older adults to test, implement, and further develop the recommendations and conceptual model presented in this meta-ethnography. We engaged in regular and ongoing critical discussions to reflect on our inherent biases and the partiality of individual interpretations. Thus, we believe that our interdisciplinary and international experiences and backgrounds were conducive to a more critical and reflexive synthesis of the studies across research contexts, and with respect to different methodological, conceptual, and ethical frameworks.

## Findings

### Outcome of study selection

Based on France et al. (2019), the following sections present findings of the study search and screening processes, including summary information and descriptive characteristics of the 10 included studies. Figure 1 presents the number of studies retrieved, screened, and included. We screened 1,736 studies at the title and abstract level in relation to the inclusion and exclusion criteria specified in Table 1 (1233 studies from the database search in July 2021, 212 and 291 studies from the updated searches in June 2022 and November 2023 respectively). Ten studies were included in the full-text evaluation, and these studies are shown in Table 2.

### Descriptive characteristics of included studies

The included studies were published between 2012 and 2020 and a detailed summary of individual studies is available in Supplementary Table S1. No studies published after 2020 were included in the present meta-ethnography. Together, the 10 included studies explored the experiences of approximately 153 older adults and 99 staff and managers, as well as shifting populations of other actors (e.g., community advisors and students) from a variety of countries: Australia (*n* = 1); Belgium (*n* = 1); England (*n* = 1); Guyana (*n* = 1); Ireland (*n* = 1); Sweden (*n* = 2); and the Netherlands (*n* = 3). The included studies used a range of methods, with some studies using multiple methods including qualitative interviews: (*n* = 8); focus groups: (*n* = 4); participant observations: (*n* = 7); informal talks: (*n* = 2); dialogue meetings: (*n* = 1); co-creation of groups: (*n* = 4); workshops: (*n* = 1); and co-creation of a creative project: (*n* = 1). The authors of the included studies named the participatory research approaches in seven different ways, with some studies presenting more than one type of participatory research approach: Action Research (AR) (*n* = 3); Appreciative Inquiry (AI) (*n* = 1); Community-Based Action Research (CBAR) (*n* = 1); Participatory Action Research (PAR) (*n* = 2); a participatory approach (*n* = 2); a participatory design approach (*n* = 1); and a collaborative and dialogical approach (*n* = 1).

### Outcome of relating studies

The outcome of relating the studies revealed characteristics that enable participatory research approaches in LTCFs for older adults, in addition to ways that researchers, older adults, staff, and other actors in LTCFs addressed participatory research approaches. Some characteristics were reported by specific groups within the LTCFs (e.g., the staff or older adults) and we recorded the sources of all quotes; however, we discerned a pervasive complexity, fluidity, and interdependence between the different actors in the LTCFs which prevented us from artificially dividing the characteristics according to discrete groups, such as staff, management, and older adults.

### Outcome of translation

The following section presents the findings of the translation process. Translation is a defining feature of meta-ethnographies (i.e., Phase 5) and the translation process involved comparing concepts *between* and *within* studies (France et al., 2019; Sattar et al., 2021). We found mainly reciprocal translations based on similarities and agreements in conceptualizations and uses of participatory research approaches, but we also identified instances of refutational translation which are presented as disconfirming cases in the findings.

### Outcome of synthesis and interpretative processes

The following sections also present our interpretation of the data from the 10 studies developed through the synthesis process (i.e., Phase 6) (France et al., 2019). First, we present (i) the outcome of the synthesis process and next, we present (ii) the outcome of the interpretative process.

#### Outcome of synthesis process

**Figure 2.**
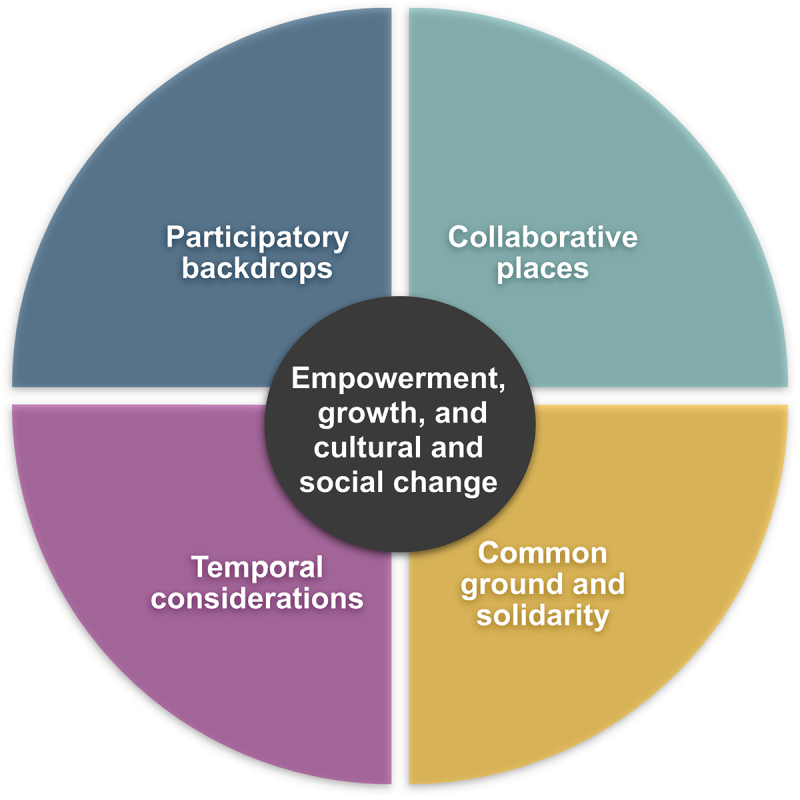
A conceptual model of participatory research approaches in LTCFs for older adults.

This section presents our synthesis of the 10 studies, with supporting quotes from the included studies. The outcome of our synthesis process and interpretations are expressed as five, third-order constructs (*Participatory backdrops*; *Collaborative places*; *Common ground and solidarity*; *Temporal considerations*; and *Empowerment, growth, and cultural and social change*), presented in a conceptual model (Figure 2).

### Participatory backdrops

*Participatory backdrops* encompass *institutional hinders*, *organizational and power dynamics*, and the *fragility, rigidity, and fluidity of participatory research approaches*. The studies showed a routinization of caring relationships in LTCFs, whereby managers were viewed as disconnected from everyday life due to rigid *organizational and power dynamics* (Baur & Abma, 2012; Hewitt et al., 2013; Mondaca et al., 2019, 2020; Willis et al., 2018; Woelders & Abma, 2019). Thus, we found *institutional hinders* to older adults’ and staff members’ opportunities to engage in participatory research approaches (Baur & Abma, 2012; Buckley et al., 2018; Hewitt et al., 2013; Mondaca et al., 2019, 2020; Snoeren et al., 2016; Woelders & Abma, 2019), to take risks, or to challenge themselves (Hewitt et al., 2013; Mondaca et al., 2019; Willis et al., 2018), as illustrated below:

Well at least for me I have to adapt myself to the situation here because where I used to live … I could please myself, do as I liked but you can’t do that here. You have to abide by the rules, right, you have to abide by the rules. (Older adult quote, (Hewitt et al., 2013, p. 11))

One study described ways in which staff performed activities in a “clandestine manner” to work around the routines and institutional hinders of the LTCF (Mondaca et al., 2020) and to better respond to the spontaneous needs and desires of older adults:

Performing activities as and when they emerge according to the residents’ pace and desires is a norm-deviating response of the staff. According to the staff, many of them resolve the dilemmas that emerge on a daily basis by acting in a “clandestine manner”. For example, they buy coloured napkins or newspapers with their own money or use their free time to fulfil the resident’s wishes, if there was no time to do so during their regular working hour. (Primary author quote, (Mondaca et al., 2020, p. 683))

However, there was a disconfirming case that showed that positive changes and empowerment of older adults were attainable when it was possible to harness the hierarchical power of the institution to foster supportive relationships with management, who in turn could help influence other actors in the LTCF to engage in the participatory research project (Woelders & Abma, 2019).

Engagement and influence were conceptualized based on the fragility (Hewitt et al., 2013; Mondaca et al., 2019; Snoeren et al., 2016; Willis et al., 2018) and contingency on the conditions of the moment (Mondaca et al., 2019; Willis et al., 2018), and on other people at various levels of the LTCF hierarchies, to be realized (Baur & Abma, 2012; Hewitt et al., 2013; Mondaca et al., 2019). Fragility and rigidity related to a “diverse palette of assumptions” (Mondaca et al., 2020). Different “palettes of assumptions” emerged from the studies, including assumptions about “knowing” the older adult (Mondaca et al., 2020) which was thought to help staff to prevent and/or manage older adults’ behaviors that were considered challenging. Assumptions, preconceptions, stigma, and stereotypes, such as the certainty of “knowing” older adults even without consulting with older adults (Hewitt et al., 2013; Woelders & Abma, 2019), could inhibit participation and serve as a resisting force for change (Buckley et al., 2018; Mondaca et al., 2020; Van Malderen et al., 2017; Willis et al., 2018).

However, assumptions, preconceptions, stigma, and stereotypes could also be contested and explored through the participatory research approaches (Buckley et al., 2018; Mondaca et al., 2020; Willis et al., 2018). Another “palette of assumptions” was revealed in Willis et al. (2018) where heteronormative assumptions about older adults living in LTCFs, and a lack of recognition of older lesbian, gay, bisexual, transgender (“LGBT”) older adults in LTCFs, inhibited the efforts of Community Advisors (CAs) who identified as “LGBT” or “LGBT” allies from using a community-based action research project to address the invisibility of “LGBT” older adults (Willis et al., 2018). The fragility and rigidity of assumptions, preconceptions, stigma, and stereotypes in LTCFs were highlighted through the participatory research approaches and may mirror the dominant preconceptions in our society, as shown in the following quote:

Some staff members claimed not to have any “LGBT” residents in their care home and showed a lack of recognition of cues indicating sexual and/or gender difference. (Primary author quote, (Willis et al., 2018, p. 9))

### Collaborative places

*Collaborative, inclusive, and safe places* were identified as enabling participatory research approaches in LTCFs. The participatory research approaches involved physicality and materiality, epitomized by the co-creation of places (Van Malderen et al., 2017) or valued objects (Pappne Demecs & Miller, 2019), as well as symbolic, temporal, and social aspects that characterized collaborative, inclusive, and safe meeting places (Mondaca et al., 2019; Van Malderen et al., 2017) and common areas (Mondaca et al., 2019).

Despite good intentions, co-creation of places was a “gradual process of maturation” (Van Malderen et al., 2017), evoking memories of everyday life before moving to the LTCFs (Baur & Abma, 2012; Buckley et al., 2018; Hewitt et al., 2013; Mondaca et al., 2019; Pappne Demecs & Miller, 2019; Woelders & Abma, 2019), as well as hopes and wishes about the potentiality of places (Mondaca et al., 2019). Thus, places and objects typically served as a participatory focus (Baur & Abma, 2012; Buckley et al., 2018; Pappne Demecs & Miller, 2019), stimulating sensory and social responses, and simultaneously the *materiality of creative expressions* contributed new qualities to the LTCF environments (Pappne Demecs & Miller, 2019). *Collaborative places* could be co-created in various ways. On the one hand, a co-created artwork could become a valued object that connected older adults who did not usually meet through the activity of co-creation and exchanging stories (Pappne Demecs & Miller, 2019). On the other hand, a disconfirming case demonstrated how places, objects, and even people involved in the participatory research approaches could also be a focus of disruption (Pappne Demecs & Miller, 2019):

… The introduction of the loom positively disrupted the routines of aged care, engaging residents in a very different way from the other leisure occupations (bingo, cards) and creative practices on offer. (Primary author quote, (Pappne Demecs & Miller, 2019, p. 111))

### Common ground and solidarity

The majority of studies using participatory research approaches enabled participants to choose topics that they perceived as *dignifying and meaningful* (Baur & Abma, 2012; Buckley et al., 2018; Hewitt et al., 2013; Mondaca et al., 2019, 2020; Snoeren et al., 2016; Willis et al., 2018; Woelders & Abma, 2019). For participating older adults, this was related to broadening choices beyond the status quo (Baur & Abma, 2012; Buckley et al., 2018; Mondaca et al., 2020; Van Malderen et al., 2017; Woelders & Abma, 2019) and the desire to be “seen” as human beings (Baur & Abma, 2012; Hewitt et al., 2013; Mondaca et al., 2019; Willis et al., 2018; Woelders & Abma, 2019), and for staff, it reflected a desire to be able to provide what they perceived as good care (Buckley et al., 2018; Mondaca et al., 2020). Perceptions of good care related to a sense of *activities giving meaning*, since exploring and adapting to the older adults’ interests and needs became more important (Baur & Abma, 2012; Buckley et al., 2018; Mondaca et al., 2019, 2020; Pappne Demecs Miller, 2019; Snoeren et al., 2016; Willis et al., 2018; Woelders & Abma, 2019), as shown below.

It should be ok to set aside the tasks in favour of doing something meaningful with the resident, this would surely improve their quality of life… .not sure we are doing that now… . (Staff quote, (Buckley et al., 2018, e862))

Through the participatory research approaches, the older adults revealed their desires for *open communication and feeling informed* about news and activities relevant to the LTCF community (Baur & Abma, 2012; Buckley et al., 2018; Hewitt et al., 2013; Mondaca et al., 2019, 2020; Pappne Demecs & Miller, 2019; Snoeren et al., 2016; Van Malderen et al., 2017; Willis et al., 2018; Woelders & Abma, 2019), to know about other older adults (to show respect and compassion) (Woelders & Abma, 2019), and to experience more personal involvement with each other as well as more engagement and connection (Hewitt et al., 2013; Woelders & Abma, 2019).

However, there were disconfirming cases where open communication was sometimes constrained by one-sided communication from the management and staff (Buckley et al., 2018; Hewitt et al., 2013; Woelders & Abma, 2019). In the case of staff, discussions about activities other than care tasks may be regarded as “taboo” (Mondaca et al., 2020) which inhibited meaningful interactions with older adults (Buckley et al., 2018; Snoeren et al., 2016). Open communication was also restricted due to privacy and ethical concerns reported by some staff members (Hewitt et al., 2013; Woelders & Abma, 2019).

Participatory research approaches evoked conversations as a stimulus for sharing memories, narratives, and desires, including exchanging narratives (Baur & Abma, 2012; Buckley et al., 2018; Hewitt et al., 2013; Mondaca et al., 2019, 2020; Pappne Demecs & Miller, 2019; Snoeren et al., 2016; Van Malderen et al., 2017; Willis et al., 2018; Woelders & Abma, 2019) and reminiscing about memories that were personal, culturally embedded, and emotional (Baur & Abma, 2012; Mondaca et al., 2019; Pappne Demecs & Miller, 2019; Willis et al., 2018; Woelders & Abma, 2019), as shown below:

First the conversation was about the weaving and about the way it is done, its colours, and the pattern … but soon residents started to talk about their craft experiences. Lorna was very keen to tell me about her knitting … Beryl also took an opportunity to tell me about how young she was when she started to make clothes for the soldiers in the war. (Field notes by the researcher, (Pappne Demecs & Miller, 2019, pp. 105–6))

However, there was a disconfirming case that revealed that sharing personal experiences may involve reliving “narratives of exclusion” (Willis et al., 2018). This is illustrated in the following quote by a CA volunteering in a participatory research project at a LTCF involving CAs who identified as “LGBT” or “LGBT” allies:

One thing I didn’t anticipate was how much this experience touched on (my) painful memories of homophobia. For me, it was about being mindful of that personal impact. Looking after myself… The project leader is a massive support, really. You can speak to her about most things. (CA quote, (Willis et al., 2018, p. 9))

### Temporal considerations

There were a variety of potential reasons which (initially) prevented older adults from being forthcoming with their opinions, complaints, and ideas for change. *Initial hesitancy* to participate was also linked to creative anticipation and curiosity towards the participatory research (Pappne Demecs & Miller, 2019), as shown below:

What is this? Is this a loom? Why are you here? What are you doing? (Resident quote, (Pappne Demecs & Miller, 2019, p. 104))

The reasons for initial hesitancy tended to be based on a sense of feeling powerless and uncertain about the consequences of participating in the participatory research approaches (Baur & Abma, 2012; Hewitt et al., 2013; Mondaca et al., 2019, 2020; Pappne Demecs & Miller, 2019; Snoeren et al., 2016; Van Malderen et al., 2017; Willis et al., 2018; Woelders & Abma, 2019). However, as the following quote demonstrates, there was a disconfirming case that inhibited older adults from initially participating, based on perceived cultural norms regarding etiquette, appropriate behaviors, and gratitude among some older adults who did not wish to disturb the status quo of the everyday activity of mealtimes at the LTCF (Baur & Abma, 2012):

Mrs Janssen stated that she felt bad complaining about the food because it was “not chic”. She referred to the way she was raised in a respected middle-class family and how she raised her own children. Cultural norms of that time led to people not complaining about food and being grateful for what you received. These ideas remained with these women. (Primary author quote, (Baur & Abma, 2012, pp. 1066–7))

Thus, the temporality of the participatory research approaches, characterized by *initial hesitancy*, may be attributed to perceived cultural norms inhibiting or delaying initial participation. As illustrated above, Mrs Janssen did not wish to appear ungrateful or to disturb the status quo in the everyday activity of mealtimes at the LTCF, and this type of *initial hesitancy* related to cultural norms may influence the timeline and process in which participatory research approaches may occur in LTCFs for older adults. *A sense of stagnation* and chaos (Baur & Abma, 2012; Buckley et al., 2018; Hewitt et al., 2013; Mondaca et al., 2019, 2020; Van Malderen et al., 2017; Willis et al., 2018; Woelders & Abma, 2019) could occur when people started to engage in participatory research approaches but there was a lack of belief that the organization would change (Van Malderen et al., 2017; Willis et al., 2018), and this may descend into negative criticisms (Baur & Abma, 2012; Hewitt et al., 2013), apathy (Van Malderen et al., 2017) and limited communication (Hewitt et al., 2013; Van Malderen et al., 2017; Willis et al., 2018; Woelders & Abma, 2019) which were unproductive for participation. Sometimes chaos emerged due to conflicting opinions and the desire for everyone to have a say (Woelders & Abma, 2019) but this chaos may also be part of the creative process if a facilitator was able to negotiate the enthusiasm and various viewpoints (Baur & Abma, 2012; Mondaca et al., 2019; Willis et al., 2018; Woelders & Abma, 2019). *Turning points* may then occur in relation to the evolving needs, motivations, and self-confidence of participants (Baur & Abma, 2012; Buckley et al., 2018; Hewitt et al., 2013; Mondaca et al., 2019, 2020; Pappne Demecs & Miller, 2019; Snoeren et al., 2016; Van Malderen et al., 2017; Willis et al., 2018; Woelders & Abma, 2019). The following quote describes such a turning point:

For Beryl, who previously rarely left her room, the novelty of the loom fostered new social connections – not only with the artist, but also with fellow residents and staff; she was “making new friends, as it is happening”. (Primary author quote, (Pappne Demecs & Miller, 2019, p. 107))

As the researchers involved in the participatory research approaches became more familiar with the social, organizational, and environmental aspects of the LTCF, they adapted and *changed* their approaches to meet the needs of the LTCF community (Hewitt et al., 2013; Willis et al., 2018; Woelders & Abma, 2019). In the following quote, a researcher reflected on the limitations of the intended participatory research approach and the need to adapt over time in collaboration with CAs volunteering in a LTCF as part of a participatory research project:

Early on in the process, CAs raised important concerns about the need for more rudimentary conversations with care staff and managers about individual beliefs, values and human rights as a necessary precursor to implementing the assessment tool … In retrospect, a more authentic co-production approach would have involved CAs in the development of such a tool… (Primary author quote, (Willis et al., 2018, p. 6))

The participatory research projects were characterized by their slow-paced progression and the perceived importance of waiting for the “right time” to set them in motion (Mondaca et al., 2020; Pappne Demecs & Miller, 2019; Snoeren et al., 2016). However, participating staff (Buckley et al., 2018; Hewitt et al., 2013; Mondaca et al., 2020; Woelders & Abma, 2019) and volunteers (Willis et al., 2018) acknowledged that they were under pressure to complete tasks due to time constraints (Baur & Abma, 2012; Buckley et al., 2018; Hewitt et al., 2013; Mondaca et al., 2020; Van Malderen et al., 2017; Willis et al., 2018; Woelders & Abma, 2019). This limited time to just sit and talk to older adults, as a way of gaining information that could inform the care that older adults received as part of the everyday routine at the LTCFs but also for the participatory research project (Buckley et al., 2018; Mondaca et al., 2020; Willis et al., 2018). Participating older adults, staff, and volunteers expressed and enacted their agency by choosing when and how they wanted to participate (Baur & Abma, 2012; Mondaca et al., 2019; Pappne Demecs & Miller, 2019), and in the following quote an older adult expressed a sense of loss at the end of the project (Hewitt et al., 2013; Pappne Demecs & Miller, 2019):

I do appreciate both of you … and I am very much sorry, sorry of losing you. (Older adult quote, (Hewitt et al., 2013, p. 12))

However, in the majority of studies, it was unclear what happened after the participatory research projects had ended at the LTCFs, in terms of *future plans and sustainability* of changes. Only one disconfirming case explictly described the consequences of the participatory research approach in the LTCF, in terms of the sustainability of the changes experienced during the project and future possibilities (Snoeren et al., 2016), as described below:

A self-sustaining mechanism came into being, expressed in the project by continuous dialogue, giving and receiving feedback and a growing professionalism based on new values and norms. It was no longer the norm to focus on “getting the day’s work done” or on the number of residents washed, but instead on the satisfaction of the resident. … The changed norms and values appeared to have become internalised which contributed to the sustainability of the changes. (Primary author quote, (Snoeren et al., 2016, p. 364))

### Empowerment, growth, and cultural and social change

Through the participatory research approaches, a growing activist attitude replaced a sense of apathy (Baur & Abma, 2012), and it was progressively supported and reinforced by the strength of the groups of fellow participants through *relational empowerment* (Baur & Abma, 2012; Mondaca et al., 2019, 2020; Pappne Demecs & Miller, 2019; Snoeren et al., 2016; Willis et al., 2018; Woelders & Abma, 2019). The participants gained a sense of pride and ownership since they were doing it for the greater good of the LTCF community and this involved the formation of a group identity, as shown by the creation of a book club (Mondaca et al., 2019) or a group of participants working to improve food quality at the LTCF, known as the “Taste Buddies” (Baur & Abma, 2012). This sense of relational empowerment and pride is demonstrated in the following quote:

If the other residents say that the meals have improved a bit, I think: ha, that’s what we achieved!

We can pat ourselves on the back. Look what we’ve managed to get done. (Older adult quote, (Baur & Abma, 2012, p. 1070))

Engagement in the participatory research approaches also provided the circumstances for the “germ of individual empowerment” (Hewitt et al., 2013) and personal growth (Hewitt et al., 2013; Pappne Demecs & Miller, 2019; Snoeren et al., 2016; Willis et al., 2018), including opportunities for learning, as participants gained self-confidence and were better able to express themselves and what they were doing. Participatory research approaches provided opportunities for the involved persons, particularly staff, to recognize older adults’ potential instead of focusing on limitations (Baur & Abma, 2012; Mondaca et al., 2019; Snoeren et al., 2016). Opportunities for *recognizing the resourcefulness of participants* also helped staff to become increasingly aware of their own actions influencing the expressions and responses of older adults (Mondaca et al., 2019, 2020; Woelders & Abma, 2019), as shown below:

For me the most surprising of all was to see that they are more together, that they light up together, the way they grow together. I didn’t expect to see this kind of support for each other here [at the NH]. (Staff (nurse assistant) quote, (Mondaca et al., 2019, p. 447))

Over the course of the participatory research projects, engagement and influence fluctuated and resulted in some *changes in relationships and roles* in the LTCFs due to increased attention to the everyday life, roles, and relationships of the older adults (Baur & Abma, 2012; Hewitt et al., 2013; Mondaca et al., 2019; Pappne Demecs & Miller, 2019; Snoeren et al., 2016; Woelders & Abma, 2019). Furthermore, *changes in values and culture* were attributed to increased opportunities for collective involvement, for example, in resident council meetings within the LTCF and a “we” feeling that would not have previously occurred at existing meetings (before the participatory research project) but which required time to develop. This sense of *changes in values and culture* is described below:

I’m positive about the meeting. We, the residents and myself, worked together and joined together instead of my former experiences with the resident council. It was not just a question and answer game. (Manager quote, (Woelders & Abma, 2019, p. 537))

#### Outcome of interpretative process

This section presents our interpretations of the third-order constructs as reviewers and our novel conceptualization (line-of-argument). The following interpretations are interwoven with, and build upon, our synthesis described in the previous section. Thus, integrating the findings from the studies into a higher conceptual level (Sattar et al., 2021).

We interpreted *participatory backdrops* as comprising the *institutional hinders*, *organizational and power dynamics*, and the *fragility, rigidity, and fluidity* of each LTCF. Together these aspects coalesce and co-constitute *participatory backdrops* to the participatory research approaches in LTCFs. *Participatory backdrops* encompass more than the physical environment and may be imbued with a latent routinization of caring relationships in LTCFs based on rigid routines, rules, attitudes, assumptions, hierarchies, and power dynamics. A possible interpretation is that an effect of this routinization was that the LTCF tended to feel more like an institution than a home. Thus, *participatory backdrops* had the potential to evoke, and even exacerbate this institution feeling as participatory research approaches were opportunities for various actors, including the older adults and staff, to question and contemplate their living or work situations respectively, as well as the underlying relationships, hierarchies, and power dynamics of LTCFs. Additionally, through the process of engaging in participatory research approaches, participants could not only be influenced by the existing *participatory backdrops* but also shape and interact with them in a more dynamic way by challenging individual and collective assumptions and attitudes in LTCFs.

Through our interpretations, the potentiality of *collaborative places* as enabling participatory research approaches in LTCFs emerged in various ways. The potentiality of *collaborative places* created shared spaces, gaps, and interludes in the everyday life of the LTCFs, by evoking creative curiosity, and providing increased opportunities for collaboration and communication that utilized but also enriched the environment and materiality of the LTCFs.

Our interpretation of the included studies showed that they provided opportunities to meet and to seek “common ground” beyond the norms, routines, and practices within the LTCFs in which participants may be reluctant to discuss activities. Thus, we interpreted participatory research approaches as contributing to “good care” but also a broader understanding of activities in the LTCFs and a sense of *activities giving meaning*, interwoven with the older adult’s interests, memories, identity, and needs. Whilst participatory research approaches could have a “humanizing” quality as people may feel “seen” and “heard”, we also interpreted challenges associated with seeking *common ground and solidarity* through engaging in participatory research approaches in LTCFs. Specifically, that traumatic memories raised potential ethical issues and underlined the importance of providing appropriate support to persons involved in the participatory research approaches, especially minority communities in LTCFs who may be at risk of stigmatization and marginalization.

We interpreted the trajectories of the participatory research approaches in LTCFs as *temporal considerations* that had the potential to enhance, secure, unsettle, or compromise a project. Despite the range of methodological and theoretical approaches of the included studies, we discerned a general, albeit non-linear and dynamic trajectory for participatory research approaches, which was characterized by *initial hesitancy, turning points, stagnation and/or change*. There was a sense that participatory research approaches bring unique risks and opportunities, as those involved “navigate unchartered waters” utilizing creative approaches that may evoke complex and at times contradictory feelings which require careful negotiation while working within the temporal, ethical, legal, social, and organizational boundaries and responsibilities of research and LTCFs. Thus, in terms of *temporal considerations*, we interpreted the participatory process of imagining, designing, discussing, scrutinizing, piloting, and implementing ideas as potentially just as important as the research outcomes for other more conventional (non-participatory) approaches to research in LTCFs.

According to our interpretations, the transformative potential of participatory research approaches could be simultaneously realized at multiple levels, through increased confidence and personal growth, as individuals and social groups, as well as through the cultivation of a more relational sense of engagement and empowerment among the broader LTCF community. This multilevel interpretation of *empowerment, growth, and cultural and social change* through engagement in participatory research approaches in LTCFs underscored the opportunities that participatory research approaches provide to recognize the resourceful dimensions of participants, which could easily pass unseen in the everyday life and routinization of the LTCFs.

Having considered the syntheses and interpretations of each of the third-order constructs, these were brought together to construct our novel conceptualization, or overarching line-of-argument. Thus, we conceptualize participatory research approaches in the context of LTCFs for older adults as: experiences of seeking common ground and social change through participation in diverse research approaches, situated in the evolving participatory backdrops and collaborative places of LTCFs. We discuss our interpretations and conceptualization in more detail in the subsequent section.

## Discussion

### Summary of findings

This meta-ethnography aimed to provide an increased understanding and novel conceptualization of participatory research approaches in LTCFs for older adults. We identified the following third-order constructs: 1) participatory backdrops, 2) collaborative places, 3) seeking common ground and solidarity, 4) temporal considerations, and 5) empowerment, growth, and cultural and social change. The findings contribute to the understanding about: a) characteristics of participatory research approaches that enable participation of older adults, staff, and other actors in LTCFs for older adults, b) ways that researchers, older adults, staff, and other actors address participatory research approaches in LTCFs for older adults, and c) conceptualizations of participatory research approaches in LTCFs for older adults. We discuss the findings in relation to these contributions in the following section.

### Characteristics of participatory research approaches that enable participation of older adults, staff, and other actors in LTCFs for older adults

Increased opportunities for collaboration and communication through participatory research approaches facilitated new ways of understanding and valuing activities in everyday life at the LTCFs. The findings contribute to a lack of knowledge regarding opportunities for innovative, non-pathologizing, and mutually empowering interactions with older adults and the environments and communities of which they are a part of (Benjamin-Thomas et al., 2018); Blair & Minkler, 2009; Smith et al., 2017). Mondaca et al. (2019) showed how participatory research approaches and *collaborative places* provided a “third space” to enable the development of a broader understanding of activities in the LTCFs, which sometimes deviated from existing norms, routines, and practices within the LTCF’s *participatory backdrops*. The “third space” is a theoretical resource which emerged in post-colonialist research and it refers to a space for hybridity and collaboration which is distinct from the home (first space) or work (second space) (Bhabha, 1994). Authors, such as Mondaca et al. (2019), framed participatory research approaches as a “third space” in LTCFs for older adults, and our findings support earlier research concerning the need to provide opportunities, or a “third space”, to participate in activities that reflect the interests and abilities of older adults (Gustavsson et al., 2015; Mondaca et al., 2018), including creative and learning opportunities (Kydd & Fulford, 2020). These opportunities or “third spaces” may exist outside routinized activities such as self-care or organized group activities, but they also build on the everyday life and care of the LTCFs for older adults to harness existing resources and to enhance the quality of care in LTCFs for older adults (Kydd & Fulford, 2020). The included studies described how participatory research approaches provided opportunities and places for staff and other actors to consider a wider variety of activities, by exploring and adapting to the older adults’ interests, strengths, and resources (Hewitt et al., 2013; Mondaca et al., 2019, 2020; Pappne Demecs & Miller, 2019; Willis et al., 2018). This helped to address assumptions, routines, and practices regarding the prioritization of care tasks (Buckley et al., 2018), and a sense that discussions about other types of activities was “taboo” (Mondaca et al., 2020). Thus, discussions about, and participation in, activities associated with the participatory research contributed to increased opportunities for meaningful social engagement and improved interactions between the various actors in LTCFs for older adults.

In several studies, it took time for the researchers of the participatory research approaches to become aware and sensitized to the intricacy and complexity of the unique *participatory backdrops* in each LTCF for older adults (Baur & Abma, 2012; Hewitt et al., 2013; Willis et al., 2018; Woelders & Abma, 2019). Differences in the *participatory backdrops* of each LTCF reflect broader cultural and contextual differences between countries and contexts. A more traditional hierarchical structure was evident in the LTCF in the study from Guyana which may correspond to a more traditional medical model of care in LTCFs (Hewitt et al., 2013). Traditional hierarchical structures tended to be less apparent, at least initially, in the LTCFs in studies from the Netherlands, Sweden, or the UK, where policies and discourses have shifted towards psycho-social and person-centered approaches to care (Corazzini et al., 2016; Edvardsson et al., 2014; Rosengren et al., 2021; World Health Organization, 2015), and increased emphasis on autonomy, choice, and self-determination among individual older adults and staff members (Woelders & Abma, 2019). Moreover, in countries such as the Netherlands, there is a legal requirement to establish a client council to involve clients in decisions regarding their own care (Woelders & Abma, 2019) and thus, national and international ethical and legal frameworks may also influence opportunities for participatory research approaches in LTCFs for older adults. The legal requirement of a client council (referred to as a resident council), to ensure involvement of residents in decision-making processes and policy making informed Woelders & Abma’s (2019) exploration of collective involvement of residents in a formal resident council, from the perspectives of management, care professionals, and residents in the LTCF. Through participatory research approaches, the resident council became a more collaborative communicative space, eschewing previous frustrations due to a lack of influence, in favor of changes in values and culture in the LTCF, whereby the resident council meetings facilitated enhanced dialogue among the different actors in the LTCF. For the purposes of this meta-ethnography, culture may be understood as encompassing interactions and social relations between social groups, in terms of beliefs, ideas, values, and expressions as well as lived experiences (Vandenberghe, 2023). Borrowing from the original authors’ (Woelders & Abma, 2019) use of language and emphasis on collective involvement, we interpreted this finding as changes in values and culture in the LTCF; however, it may be interpreted in various ways, including in relation to changes in attitudes and perceptions among actors in the LTCF.

Despite cultural and contextual differences, the view of the LTCF as both a home and institution, sometimes referred to as the “home paradox”, permeated conceptualizations of the *participatory backdrops* across the included studies. Authors, such as Martin (2002), have pointed to the paradoxical situation of LTCFs for older adults which simultaneously function as homes for older adults as well as places to work for other actors, such as staff and management, each with their own respective interests, practices, and values (Artner, 2018). Across the studies, we identified a continuum between the view of LTCFs as a home or an institution, and this seemingly influenced opportunities for participation in participatory research approaches. The “home paradox” has been discussed in relation to care for older adults in general and in LTCFs specifically (Artner, 2018; Cutchin et al., 2003; Mondaca et al., 2020), but scarcely as an aspect of *participatory backdrops* for participatory research approaches in LTCFs for older adults. Through engagement in the participatory research, older adults conveyed a sense of agency and ownership over their everyday lives since the LTCF was considered their home, for example through the co-creation of a group of older adults who came to be known as the “Taste Buddies” (Baur & Abma, 2012), and a book club (Mondaca et al., 2019), as well as the co-creation of artworks to contribute to the materiality of the LTCF (Pappne Demecs & Miller, 2019). This view sometimes collided and contradicted with staff and management, who tended to view the LTCF as an institution due to its paid staff, administrative and regulatory structures about visitation, access to different areas, and routines about meals, medication, and personal care (Artner, 2018; Martin, 2002). Thus, the findings highlight the importance of considering *participatory backdrops* with respect to the “home paradox” in LTCFs for older adults.

### Ways that researchers, older adults, staff, and other actors address participatory research approaches in LTCFs for older adults

The potential for participatory research approaches to contribute to social engagement and community-building has been discussed in earlier literature (Benjamin-Thomas et al., 2018; Blair & Minkler, 2009; Burns et al., 2021; Scheffelaar et al., 2020; Shura et al., 2011) but there is limited research problematizing communities and how this relates to the fragility, rigidity, and fluidity of participatory research approaches in LTCFs. The findings elucidated a sense of fragility since participatory research approaches are contingent on the conditions of numerous other actors, or members of the LTCF community, to be realized. This finding corroborates earlier research regarding ageist assumptions about the frailty and capacity of older adult communities living in LTCFs (Gustavsson et al., 2015; Mondaca et al., 2018), and decisions by other actors, namely staff and management in positions of power and authority, about what constitutes appropriate activities for older adults (Fallahpour et al., 2022; Gustavsson et al., 2015; Wood et al., 2009). The findings revealed different “palettes of assumptions” including mundane assumptions about everyday activities but also preconceptions, stigma, and stereotypes concerning gender, identity, and community. There were instances of participatory research approaches contributing to positive community-building actions, such as the co-creation of the “Taste Buddies” group (Baur & Abma, 2012), or a book club (Mondaca et al., 2019), but there was also an example of exclusion based on assumptions, preconceptions, stigma, and stereotypes among different actors in the LTCF (Willis et al., 2018). Earlier research on stigmatizing discourses (Manchha et al., 2023) has identified stigma in care contexts for older adults. Stigma may be defined in various ways and according to Goffman (1963, p. 3) it is “an attribute that is deeply discrediting” that can devalue one’s social identity in a given social context. In Willis et al.'s (2018) study, CAs identifying from one community, the “LGBT” community, encountered hostility from members of another community (some staff members of the LTCF community) during a participatory research project (Willis et al., 2018). This finding aligns with earlier research indicating that specific communities in LTCFs, such as older adults identifying as “LGBT”, may face increased stigma (Rosenberg et al., 2018) which exacerbates epistemic injustices (Fricker, 2007, 2013), and limits opportunities for participation based on intersectional aspects, such as ethnicity, sexual and gender identity (Rosenberg et al., 2018). This meta-ethnography’s findings highlighted that older adults are a heterogenous group. To avoid one-sided statements, further research is needed to explore increased opportunities to involve heterogeneous groups of older adults in participatory research approaches and how this relates to heterogenous members of staff and other actors in LTCFs for older adults.

Our findings indicate that participatory research approaches have potential to address epistemic injustices by co-creating *democratic spaces of exchange and collaboration* where older adults may mediate knowledge production and various actors in LTCFs can learn from each other. Thus, helping to democratize knowledge making processes by highlighting older adults’ heterogeneous voices and opportunities for older adults’ active involvement in research (Groot et al., 2022). The present meta-ethnography’s conceptualization of participatory research approaches provides new perspectives on challenges, as well as steps towards collaborative problem solving in LTCFs for older adults. To foster collaborative problem solving, the findings highlight the need to reflect upon moments of fragility such as reliving “narratives of exclusion”, the rigidity of assumptions, preconceptions, stigma, and stereotypes among actors in LTCFs for older adults, but also fluidity as members of different communities come together through participatory research in LTCFs for older adults.

Previous research has emphasized the importance of transparent reporting in publications, ethical and funding applications, particularly regarding preparatory stages in participatory research approaches with older adults (Hand et al., 2019); however, our findings suggest that there is a need for increased planning and clearer reporting across all stages of participatory research. Across the included studies, we discerned a general yet non-linear trajectory of initial hesitancy, turning points, stagnation and/or change over the course of the participatory research approaches. Initial hesitancy was a reoccurring consideration in the included studies, especially among staff and older adults as they adjusted to the new project entering their workplace or home. Earlier research regarding exemplars and best practices for conducting participatory research approaches advise that researchers should establish contact and develop relationships with the community where the research will take place, and this can take time (Benjamin-Thomas et al., 2018; Blair & Minkler, 2009; Hand et al., 2019). Some of the included studies described allocating time to familiarize themselves with the environment and actors in the LTCFs (Mondaca et al., 2019), whereas other studies reported having insufficient time which created challenges such as having to change their original study design or approach as they became more aware of the local needs, resources, and preferences (Hewitt et al., 2013; Willis et al., 2018). Few of the included studies described the end of participatory research projects, thus future research would benefit from a greater consideration for sustainability and future plans of the participatory research once the researchers have left and a project is formally completed in the LTCF. Furthermore, there was a tendency to emphasize flexibility among the various participatory research approaches identified in the included studies, of which some studies such as Baur and Abma (2012), used a “blended approach” combining different yet overlapping participatory research approaches. Such flexible and blended approaches may be well-suited to the non-linear trajectories of performing participatory research approaches in LTCFs for older adults.

### Conceptualizations of participatory research approaches in LTCFs for older adults

Based on the findings of this meta-ethnography, we conceptualize participatory research approaches in the context of LTCFs for older adults as: experiences of seeking common ground and social change through participation in diverse research approaches, situated in the evolving participatory backdrops and collaborative places of LTCFs. Our conceptualization is a subtle departure from generic guidance on participatory research approaches (Benjamin-Thomas et al., 2018; Blair & Minkler, 2009; Burns et al., 2021; Reason & Bradbury, 2001, 2008; Scheffelaar et al., 2020) which emphasizes the method and design procedures rather than action and social change.

To support the design and implementation of participatory research approaches in LTCFs for older adults, we integrated our conceptualization and third-order constructs into six recommendations. The recommendations are targeted towards researchers of participatory research approaches, older adults themselves, as well as the wider community of actors involved in participatory research approaches (i.e., staff and management, policymakers, funders, and members of ethical boards for research), since these actors are somewhat overlooked in earlier research and textbooks on participatory research approaches (Blair & Minkler, 2009; Burns et al., 2021; Reason & Bradbury, 2001, 2008; Scheffelaar et al., 2020). The recommendations are shown in Table 5.

**Table 5.** Recommendations for participatory research approaches in LTCFs for older adults.

No.	Recommendation	Target group
1.	Policies regarding care and everyday life in LTCFs for older adults, should encourage the design and implementation of participatory research approaches as opportunities for improvement of LTCFs and for empowerment and growth among persons living and working in LTCFs.	Policymakers
2.	During participatory research approaches, recognize and work with the resourcefulness of different participating persons and remain open to the instability of the approach, including initial hesitancy, turning points, stagnation and/or change.	LTCF managers, staff and other actors
3.	Participatory research approaches should build on everyday life and care in LTCFs for older adults.	LTCF managers, staff, other actors and older adults
4.	Opportunities to engage in participatory research approaches should be supported in order to access increased understanding of the meaning of activities and ways of working in LTCFs for older adults.	
5.	Promote collaborative, inclusive, and safe spaces for participatory research approaches in LTCFs to address assumptions, preconceptions, stigma, and stereotypes in the LTCF community.	Researchers of participatory research approaches
6.	When planning participatory research approaches in LTCFs for older adults, allow flexible time for the slow-paced progression before, during, and after participatory research approaches due to the emergent style of working.	Researchers of participatory research approaches and Funders/ethical boards

### Summary and clarification of the recommendations

To clarify, the six recommendations highlight the importance of integrating opportunities for older adults and other actors to engage in participatory research approaches in LTCFs for older adults. This can increase the relevance, acceptability, and usability of research to enhance care for older adults and promote their health and well-being. Given the emphasis on collaboration within participatory research approaches, there is an emphasis on the democratization of knowledge by involving different actors (Benjamin-Thomas et al., 2018; Groot et al., 2022). Thus, the recommendations are tailored on multiple levels according to different target groups. At a policy level, there is a need for relevant policies and investment to support the design and implementation of participatory research approaches which have demonstrated potential to improve the health and well-being of older adults in LTCFs, as described in this meta-ethnography and earlier literature (Benjamin-Thomas et al., 2018; Cargo & Mercer, 2008) (Recommendation 1). Aligning with earlier literature emphasizing the importance of a supportive organizational culture in LTCFs for older adults (Backhouse et al., 2016), there is a need for support and endorsement from managers specifically, as well as other actors, in LTCFs to provide space and time for researchers to work in flexible, open, and collaborative ways with the different actors (Recommendation 2). Participatory research approaches are typically conducted in-situ within the LTCF for older adults (Benjamin-Thomas et al., 2018); thus, it is integral that managers and other actors, including older adults, recognize the need for participatory research approaches to be integrated into everyday life in the LTCFs (Recommendation 3), with clear processes for opting out if required. In keeping with the everyday life and in-situ character of participatory research approaches (Benjamin-Thomas et al., 2018; Shura et al., 2011), opportunities to engage in the research should be open and widely accessible to older adults as well as staff and other actors, thus this necessitates support from the various levels of organization at LTCFs for older adults, such as managers, staff, older adults, and other actors (Recommendation 4). To help ameliorate epistemic injustices that risk undervaluing personal narratives (Groot et al., 2022), researchers should promote collaborative, inclusive, and safe spaces for participatory research approaches which are cognizant of assumptions, preconceptions, stigma, and stereotypes in the LTCF community (Recommendation 5). Finally, researchers themselves and their respective funding and ethical boards should consider in advance the need for flexible timelines that accommodate the slow-paced and emergent character of participatory research approaches, especially when working collaboratively with multi-level actors in LTCFs for older adults, to avoid tokenistic gestures in response to grant funders’ and ethical boards’ calls for increased patient and public involvement (Benjamin-Thomas et al., 2018; Cargo & Mercer, 2008; Hand et al., 2019) (Recommendation 6).

### Strengths and limitations

We utilized systematic methods according to the eMERGe (France et al., 2019), ENTREQ (Tong et al., 2012), PRISMA (Page et al., 2021), and STARLITE guidelines (Booth, 2006), but there are potential limitations to consider. Our original intention was to include home care services in addition to LTCFs in our database search; however, a preliminary database search failed to retrieve relevant studies in the context of home care services. This motivated us to amend our protocol to focus solely on LTCFs. However, our research questions sought to explore participatory research approaches broadly, among older adults as well as staff and other actors in LTCFS for older adults. Aligned with participatory research approaches, the meta-ethnography explored interactions and relations *between* and *within* the different groups of actors in this setting. This motivated the inclusion of some studies (Mondaca et al., 2020; Willis et al., 2018) which involved older adults and other actors in LTCFs although the primary focus may have been on staff. By including such studies, it also revealed potential compromises and tensions when using participatory research approaches that highlight specific voices (i.e., staff) within LTCFs for older adults and may perpetuate epistemic injustices.

Despite searching and reviewing more recent studies, as presented in Figure 1, the most recent study to match our inclusion and exclusion criteria and to contribute relevant data to our research aim and questions was published in 2020 (see Figure 1 for reasons for exclusion and Table 1 for further details on the literature search strategy, including the inclusion and exclusion criteria). This may partly be explained by COVID-19 and quarantining regulations to protect people living and working in LTCFs which also restricted access to LTCFs among external visitors, including researchers (Gordon et al., 2020; World Health Organization, 2022). The disruption of everyday research activities has been referred to as “scholarly displacement” and is salient to participatory research approaches due to the emphasis on actions in situ and in collaboration with various actors (Auerbach et al., 2022). As our findings show, participatory research approaches typically involve group activities in *collaborative places* (i.e., common areas), which were particularly affected by changes in social, organizational, and environmental aspects of LTCFs due to COVID-19, such as isolation of older adults in their private rooms (Gordon et al., 2020; World Health Organization, 2022). Future research may build on this meta-ethnography to ascertain similarities and differences in the conceptualizations and uses of participatory research approaches before, during, and after COVID-19.

In the most recent literature search (November 2023), 22 articles were taken to full-text screening due to the perceived relevance of participatory research approaches highlighted at the title and abstract level. However, the full-text review revealed a tendency in this search (*n* = 10) and also in the previous searches, for authors to utilize keywords and terminology related to participatory research approaches despite using more conventional qualitative research methods reliant on a traditional academic researcher-research subject relationship. To avoid diluting the meaning and potential implications of participatory research approaches, the findings in this present study suggest that there is a need for future research with thorough conceptualization and use of participatory research approaches situated in the context of LTCFs for older adults. Our proposed conceptual model and recommendations may be used in future studies to consider more collaborative and balanced relations that promote the democratization of knowledge and to emphasize social action and change which are inherent to participatory research approaches in LTCFs for older adults.

The lack of more recent publications may also account for the use of “LGBT” in the publication by Willis et al. (2018); however, we acknowledge that terminology has evolved, and may continue to do so over time. Thus, for the purposes of this meta-ethnography, the authors acknowledge the more inclusive and current term of LGBTQ2S+ (lesbian, gay, bisexual, trans, queer, and two-spirit) (Lipinski et al., 2023) but use quotation marks when referring directly to the terminology used in the included primary studies.

We used an English language restriction in our database search which may limit the generalizability of the findings to different countries and contexts. The inclusion of only published, peer-reviewed articles may have excluded other relevant studies published in book chapters or “grey literature” (Malpass et al., 2009). Still, the purpose of this meta-ethnography was not an exhaustive review of all participatory research approaches in LTCFs and we recognize the partiality of our interpretations without access to primary data. Given that our research group explores participatory research approaches among older adults, it is unsurprising that our database search retrieved examples of our own research and/or associated researchers (i.e., Mondaca). Similarly, there were instances of authors being involved in more than one of the studies (i.e., Abma, Mondaca). Studies were included based on their relevance to our search criteria. It was, however, important to be transparent about such relationships and to use critical appraisal and ongoing discussions to minimize biases and promote reflexivity.

## Conclusion

Numerous articles and textbooks describe how to conduct participatory research approaches in general; however, to the authors’ knowledge, this is among the first qualitative synthesis to explore conceptualizations and uses of participatory research approaches in the context of LTCFs for older adults. There are practical implications regarding the empowerment, growth, and cultural and social change that emerged from the participatory research in LTCFs since the epistemology of participatory research approaches endeavors to improve everyday life, as opposed to merely studying it. The participatory research approaches involved a process of reacting to, and potentially transforming, existing hierarchical models, practices, and traditional monopolies of power and knowledge. This is particularly important in health and social care research where insufficient involvement of end users of health and social care research may compound health inequities among potentially marginalized communities, such as older adults in LTCFs. Participatory research approaches have potential to address epistemic injustices and to co-create *democratic spaces of exchange and collaboration* in which various actors in LTCFs can learn from each other. Thus, our conceptual model and recommendations have implications for the various actors involved in participatory research approaches, by encouraging them to consider enabling characteristics and ways to address participatory research approaches when designing and implementing participatory research approaches in LTCFs for older adults.

## Abbreviations

AI: Appreciative inquiry
AR: Action research
CA: Community advisor
CASP: Critical appraisal skills programme checklist
CBAR: Community-based action research
CBPR: Community-based participatory research
CINAHL: Cumulative index of nursing and allied health literature
ENTREQ: Enhancing transparency in reporting the synthesis of qualitative research statement
ERIC: Education resources information center
LGBT: Lesbian, gay, bisexual, transgender
LGBTQ2S+: lesbian, gay, bisexual, trans, queer, and two-spirit
LTCF: Long-term care facility
PAR: Participatory action research
PRISMA: Preferred reporting items for systematic reviews and meta-analysis statemen
PROSPERO: International prospective register of systematic reviews.

## Supplementary Material

Supplementary Table 1.docx

## Data Availability

The datasets used and/or analyzed during the current study are available from the corresponding author on reasonable request.
