# Supplementary material for: Participatory research approaches in long-term care facilities for older adults: a meta-ethnography
Source: Int J Qual Stud Health Well-being. 2024 Nov 24;19(1):2431449. doi: 10.1080/17482631.2024.2431449 (PMC11587718; doi:10.1080/17482631.2024.2431449)
Supplement: Supplementary Table 1.docx [file ZQHW_A_2431449_SM3587.docx]

**Supplementary Table 1** Descriptive characteristics of included sources (*n* = 10)

| **Authors, (year), country** | **Aim** | **Type of participatory research approach** | **Participants and setting** | **Methodology/ data collection** | **Data analysis** | **Findings related to participatory research approach** | **Conceptual/ theoretical perspectives** | **Ethical considerations** |
| --- | --- | --- | --- | --- | --- | --- | --- | --- |
| Baur & Abma, (2012), The Netherlands. | To enhance the interactive and  collective participation of residents in practice improvements and policy  issues that affect their daily life in the residential home. | Action Research (AR) including Appreciative Inquiry (AI). | 7 female residents at the residential home.  Age group:  82 – 92 years. | AR project with a group of the residential home residents (‘Taste Buddies’).  Co-creation of an action group and dialogue meetings. | Ongoing AR analysis.  The residents were involved in the qualitative analysis through member checking. | Resident participation as partnership with employees and managers starts with **relational**  **empowermen**t among residents themselves (**enclave deliberation**). | Theoretical framework based on combination of AR and AI. Social constructivist view and relational empowerment. | Consideration of research ethics in relation to research partnerships and relationships. |
| Buckley et al., (2018), Ireland. | To evaluate the effects of the implementation of a methodological  framework for a narrative-based approach to practice development and person-  centred care in residential aged care settings. | Action Research (AR) using narrative practice. | 37 residents and 38  Staff in 2 residential care settings.  Age group:  Not described by the authors. | AR using narrative practice and emancipatory practice development.  Work-based learning groups with staff, focus groups and interviews with residents, and participant observations. | Ongoing AR analysis, including thematic analysis, documentary analysis, and creative hermeneutic data analysis. | The implementation of a **framework of narrative practice** showed how people respond to change, the development of **shared understandings** and **intentional action**. | Theoretical framework based on AR and other theoretical resources such as emancipatory practice development. Framework of Narrative Practice, and person-centred nursing framework (i.e., narrative aspects of care, work-based learning sessions). | Statement of ethical approval. |
| Hewitt, Draper & Ismail, (2013), Guyana. | To analyse and improve quality of life in a residential home for older people in  Guyana. | Participatory approach using participatory needs assessment and qualitative process evaluation. | Focus group discussions (needs assessment: 12 residents and 4 staff; end of project: 14 residents and 4 staff). Semi-structured interviews (needs assessment: 2 committee members, 1 administrator and 1 volunteer nurse; end of project: 1 committee member and 1 administrator).  Age group:  73 – 99 years | Case study of a participatory approach using a qualitative process evaluation (participant observation, focus group discussions and individual semi-structured  interviews). | A framework approach to data analysis comprised of five interconnecting stages: familiarisation, identifying a thematic framework, indexing, charting, and mapping and interpretation. | The qualitative process evaluation revealed the degree of participation achieved, the determinants of the participatory process, as well as the **ethics of participations** (i.e., benefits and **ethical dilemmas** encountered) in the not-for-profit residential home. | Theoretical background framed according to ethical and practical dilemmas and using a participatory approach. | Discussion of ethical issues regarding the implications of participatory approaches used in smaller institutions (i.e., the risk of highlighting problems and generating conflicts). |
| Mondaca et al., (2019), Sweden. | To explore how older adults’ engagement and influence in an occupation can emerge in  everyday life in a nursing home setting. | Participatory approach using co-creation with engagement and influence in occupations. | 1 researcher, 1 nurse assistant, 7 residents who participated frequently and 5 residents who participated less frequently in the book club.  Age group:  74 – 90 years. | Participatory approach involving a book club co-created by the researchers,  residents, and the nursing home community.  Co-creation of a group as a ‘third space’ (Book club), qualitative interviews and participant observations. | Hermeneutic  interpretative approach. | Engagement and influence in occupations in a nursing home through the **co-creation and participation in a ‘third space’** in the form of a book club. | Theoretical background based on engagement in occupations and participatory approaches. | The authors described the use of a reflective attitude to constantly return to ethical considerations throughout the study. |
| Mondaca et al., (2020), Sweden. | To better understand how a dialogue about the influence of nursing home residents on their everyday activities evolve among diverse practitioners and to identify the consequences  of such an understanding in practice. | A collaborative and dialogical approach. | 19 nursing home practitioners (16 nursing assistants, 1 nurse, 1 occupational therapist, 1 caretaker).  Age group:  Not described by the authors. | A collaborative and dialogical approach involving 5 workshops, 1 focus group and follow-up interviews. | Analysis followed a dialogical approach | The nursing home staff described acting in a **clandestine manner** to create ways of enabling  **‘humane’ practices** towards the residents - to make sense of everyday activities for residents  institutional routines. | Theoretical background based on the conceptualisation of engagement in everyday activities, reframing everyday activities, and clandestine manners. | Discussion about the ethical aspects at stake for nursing home residents in everyday activities. |
| Pappne Demecs & Miller, (2019), Australia. | To explore if and how creative occupation, a participatory art project, might benefit older people living in residential aged care. | Participatory design approaches (co-design and co-creation). | 30 residents in a residential aged care facility (although active participation varied throughout the project).  Age group:  All ages of participants not described by the authors but 3 residents in focus aged 82-90 years. | Co-creation of a creative project: a professional tapestry artist invited residents to co-design and co-create a large tapestry project. Multiple types of data collection: researcher field notes, observations, researcher-produced photographs and videos of process, informal conversations, and post-art participation semi-structured interviews. | Qualitative case study using Interpretative phenomenological analysis, with researcher field notes and a visual essay. | The findings reveal how residents **experienced the co-design and co-creation** project and a **sense of flow in diverse ways** (gradually, partially, and fully engaged). The link between **engagement in a creative occupation** (i.e., tapestry weaving) and cognitive, physical, creative and emotional **wellbeing** among the older residents was also emphasised. | Theoretical background based on participatory design approaches (i.e., collaborative weaving, participatory art, and craft making as an experience of sharing and telling). | Ethical considerations about relations between participants. |
| Snoeren et al., (2016), The Netherlands. | To examine what has contributed to the improvement of participation of older people with dementia in daily occupational and leisure activities according to practitioners. | Action Research (AR) project transformational change processes. | A shifting population of 22 older residents in a nursing home. 1 nurse manager; 20 licensed practical nurses, registered nurses and healthcare professionals; 18 students from various care training programmes; 4 ward assistants and; 1 part-time activities coordinator.  Age group:  Not described by the authors. | A case study about an Action Research (AR) project, including a meta-study to gain additional insights about transformation change processes. Data were collected through participant observation, interviews and focus groups. | Thematic analysis. | The **Action Research (AR)** project involving **spontaneous interactions** was linked to increased participation in daily activities and a **cultural transformation** toward more person-centredness. The metaphor of a blossoming cherry tree was used to communicate the cultural transformation. | Theoretical background guided by organisation change processes and Kemmis and McTaggart's (1988) action cycle framework. | Ethical considerations discussed in relation to quality assurance. |
| Van Malderen et al., (2017), Belgium. | To discuss possible contributions of participatory action research as  structural method in a new Active Ageing-envisioned nursing home, enabling residents’  participation and focuses on the practicalities of its implementation process. | Participatory Action Research (PAR). | 9 residents.  Age group:  Mean age 85 years (SD: 6.7 years). | An Active Ageing implementation project using PAR.  Co-creation of a PAR group (audio recorded group meetings) and participant observations. | Qualitative content analysis. | The study revealed **practical insights** about the feasibility of the **implementation using PAR** and its potential to enhance the residents’ quality of life through the realisation of an Active Ageing-envisioned nursing home. | Theoretical framework based on Active Ageing and participation in the context of nursing homes. | Discussion about ethical issues in relation to change processes. |
| Willis et al., (2018), England, UK. | To pilot and evaluate a scheme within six care homes in a  large city in England that would enhance the inclusion of older lesbian, gay, bisexual and transgender (LGBT) residents. | Community-Based Action Research project using co-production. | Phase 1: action research collaboration with 1 independent housing provider and 6 of its care homes for older people, 1 project leader, 8 community advisors, and the LGBT advisory group.  Phase 2: 19 respondents (8 community advisors, 8 care home managers, and 3 key informants e.g., community engagement officer and external facilitator who led training sessions).  Age group (only provided for community advisors):  35 – 65 years. | Community-Based Action Research project involving pre- and post- project qualitative evaluation.  Qualitative interviews. | Qualitative analysis (unspecified). | A critical reflection on the **ethics and effectiveness of co-production** as a collaborative approach to **stimulate organisational change** in older people’s care environments, to use non-didactic storytelling in LGBT awareness-raising among staff, and to promote **inclusion**. | Theoretical background based on co-production (i.e., to challenge power asymmetries) as well as LGBT, sexual and gender identity. | Critical reflection on the ethics and effectiveness of using co-production as a collaborative approach to action-orientated research with older LGBT nursing home residents. |
| Woelders & Abma, (2019), The Netherlands. | To shed light on this process of involving residents in a health care organisation  by dialogue amongst all those involved. | Participatory Action Research (PAR) approach. | 10 residents in the residential care home (at the start of the project).  Age group:  67 – 95 years. | PAR approach project. Utilising  Habermas’ theory to explore the creation of communicative spaces through dialogue.  Participant observations, informal talks, semi-structured interviews. | PAR qualitative analysis in relation to Habermas’ theory. | A learning experience about the need to consider issues of power in **PAR** and the potential of dialogue to improve mutual understanding and open up **communicative spaces**. | Theoretical background based on PAR as well as Habermas’ theory with concepts such as communicative action and communicative spaces. | Question of ethical appropriateness in relation to participants’ privacy. |
